# Understanding the genetic imperfections of Lewy body dementia

**DOI:** 10.1186/s13024-026-00961-1

**Published:** 2026-07-08

**Authors:** Molly M. Watkins, Gisela Xhafkollari, Na Zhao, Owen A. Ross

**Affiliations:** 1 Department of Neuroscience, Mayo Clinic, 4500 San Pablo Road, Jacksonville, FL 32224 USA; 2 Clinical and Translational Science Graduate Program, Mayo Clinic Graduate School of Biomedical Sciences, Jacksonville, FL USA; 3 Neuroscience Graduate Program, Mayo Clinic Graduate School of Biomedical Sciences, Jacksonville, FL USA; 4 Department of Clinical Genomics, Mayo Clinic, Jacksonville, FL USA

**Keywords:** Lewy body dementia, Genetics, *SNCA*, *APOE*, *GBA*

## Abstract

Lewy body dementia (LBD) is a clinically and pathologically complex neurodegenerative disease. Its etiology remains poorly understood and, as such, it is likely underdiagnosed in clinical practice. Clinically, LBD is defined by fluctuations in cognition, visual hallucinations, rapid eye movement (REM) sleep behavior disorder (RBD), and motor dysfunction. The disease is pathologically defined by the presence of Lewy bodies in the brain, which are protein aggregates made up of misfolded α-synuclein; however, co-pathologies are often present and greatly complicate both symptom presentation and research interpretation. This heterogeneous pathology paired with overlapping clinical features of Alzheimer’s disease and Parkinson’s disease hinder diagnostic clarity. With no specific biological biomarkers currently identified, it is the hope that genetics can help with diagnosis and to clarify the pathomechanistic nature of this multifaceted disorder. Through rare familial studies, *SNCA*, the gene which encodes α-synuclein, was implicated in disease risk and development, underscoring its central role in α-synucleinopathies. Shortly after this discovery, a genome wide association study (GWAS) confirmed this risk, and identified two other major genes, *APOE* and *GBA*, to be associated with LBD. The *APOE* ε4 allele, which is a well-established risk factor for AD, is also associated with an increased risk for LBD. This common association demonstrates that *APOE* is implicated in a broader role in neurodegenerative disease pathogenesis. Meanwhile, *GBA* mutations, which are associated with lysosomal dysfunction and are also a known risk factor for PD, are linked to a more severe cognitive decline and earlier disease onset. However, even with these advances, our understanding of LBD’s genetic architecture remains incomplete. Overcoming these limitations will require larger, more diverse study cohorts, comprehensive analyses, technological advances, and improved phenotyping upon clinical presentation of disease. Current clinical diagnostic strategies often struggle to capture the full spectrum of LBD symptoms and commonly lead to misdiagnosis/misclassification. By combining genetic data with improved phenotyping and the latest novel technologies, we can greatly improve our mechanistic understanding of LBD. These insights will not only improve diagnostic accuracy but will also lead to the development of disease modifying treatments specifically designed to best treat each individual patient.

## Introduction

“Everyone you meet is fighting a battle you know nothing about. Be kind. Always,” advice from the late Robin Williams, whose own battle included the devastating effects of neurodegeneration. His post-mortem examination revealed widespread Lewy body disease, the underlying brain pathology which, unknown to him, had manifested clinically as Lewy body dementia (LBD). His struggle and subsequent diagnosis cast public spotlight on a condition that remains widely misunderstood and underrecognized, despite its profound impact on patients and their families. By 2050, an estimated 139 million people will be living with dementia worldwide [1], and LBD is estimated to account for approximately 10–15% of cases [1, 2]. Unfortunately, due to the lack of a definitive clinical diagnostic test, LBD is likely underdiagnosed, as is demonstrated in the gap between clinical diagnoses during life and the higher prevalence of Lewy body disease detected on neuropathological examination [3–6].

This diagnostic uncertainty reflects the complex clinical and pathological nature of LBD, which encompasses two overlapping syndromes: dementia with Lewy bodies (DLB) and Parkinson’s disease dementia (PDD). While both involve cognitive decline and parkinsonian features, the distinction between them lies primarily in the timing of symptom onset [5, 7]. However, this boundary is often blurred in clinical practice, contributing to underrecognition and misdiagnosis. This overlap also extends to genetic studies where many researchers include both PDD and DLB within combined cohorts of LBD, reflecting their overlapping clinical presentation. LBD also shares extensive pathological and clinical overlap with both Alzheimer’s disease (AD) and Parkinson’s disease (PD), making it difficult to delineate its unique biological underpinnings. In fact, many patients with LBD have coexisting AD pathology [8–12], and estimates suggest more than 80% of individuals with PD will eventually develop dementia [13–16], further complicating efforts to distinguish these disorders genetically and pathologically.

In recent years, genetics has emerged as a critical tool in trying to disentangle these relationships and identify risk factors that might predict or explain disease onset and progression. However, unlike AD or PD, where major genetic risk loci have been robustly identified and replicated, the genetic architecture of LBD remains less well-defined. While genes such as *SNCA*, *APOE*, and *GBA* have been identified, the field continues to face limitations, ranging from relatively small sample sizes and diagnostic variability to limited population diversity and overlapping pathology [17].

Robin Williams’ character in the movie *Good Will Hunting*, states regarding character, “People call these things imperfections, but they’re not, that’s the good stuff.” What we often see as biological imperfections (in the context of LBD, mutations in genes like *SNCA*,* APOE*, and* GBA*), while not inherently good, serve as critical clues to pathogenesis. By studying these imperfections, it is hoped that we can uncover mechanisms of disease and discover the pathways towards palliative treatments and effective therapies. In this review, we explore these “imperfections” in our understanding of LBD genetics. We examine current knowledge, recent discoveries, and persistent gaps, while proposing future directions to help clarify the genetic underpinnings of this complex and understudied disorder.

## Clinical and pathological complexity of LBD

### Clinical presentation

**Fig. 1:**
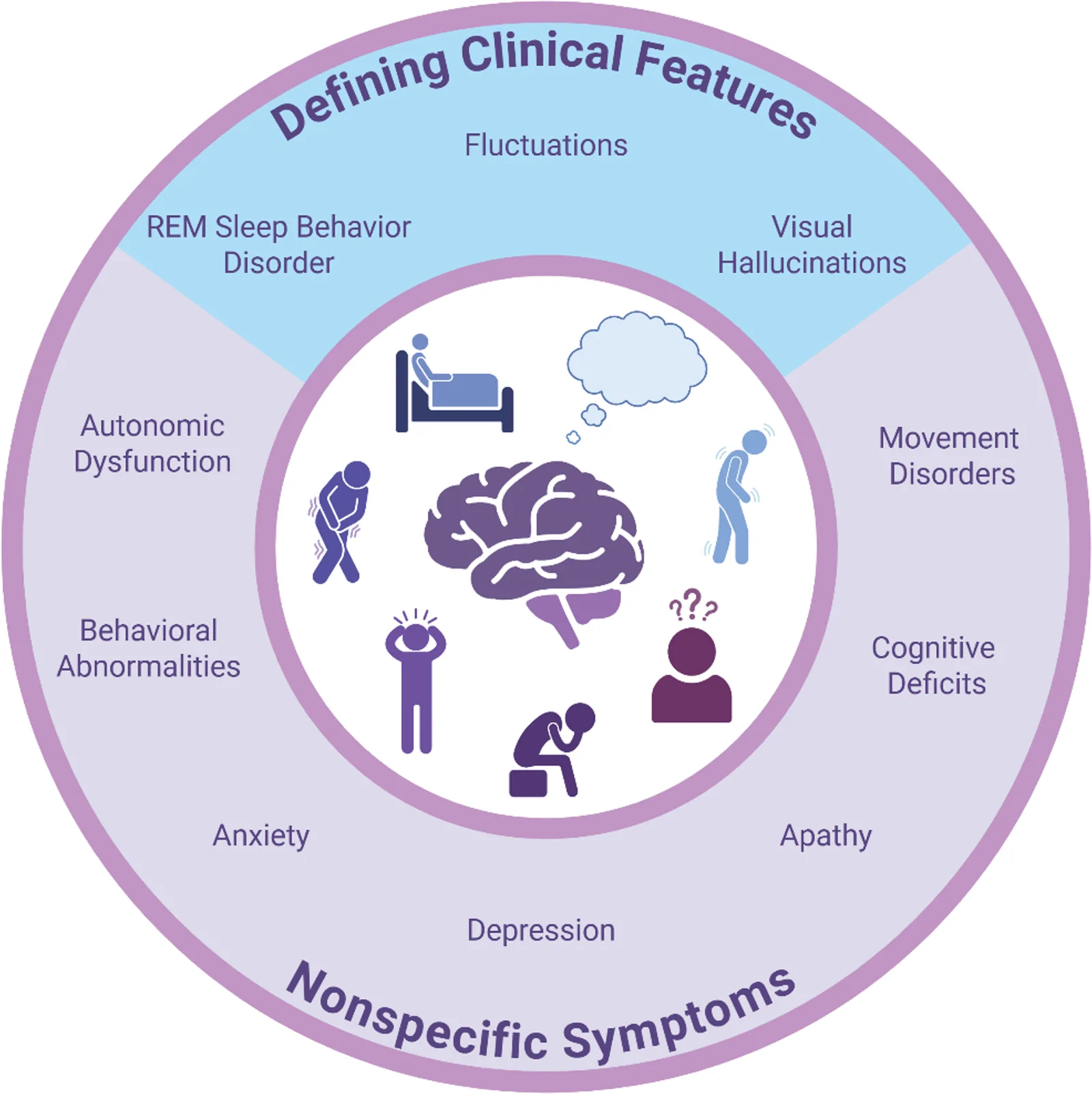
LBD clinical presentation. Summary of common symptoms associated with LBD (Created in BioRender. Watkins, M. (2026) https://BioRender.com/esaap4t)

The clinical presentation of LBD falls within the intersection of two more common neurodegenerative diseases: PD, characterized by progressive movement disorder [18], and AD, clinically defined by memory loss and behavioral changes [18–20]. With similar clinical signs across LBD, AD, PD, and neuropsychiatric disorders, especially in the early stages of disease when symptomology can often be ambiguous, it can be difficult to diagnose patients accurately. The key distinguishing symptoms of LBD include visual hallucinations, fluctuations in cognition and attention, and rapid eye movement (REM) sleep behavior disorder (RBD) [7]. Other symptoms of LBD include deficits in motor function, mood disorders, behavioral abnormalities, depression, anxiety, apathy, and autonomic dysfunction (Fig. 1) [4, 21]. Recent studies have highlighted the importance of identifying prodromal signs of LBD such as RBD, which has been recognized as a risk factor for α-synucleinopathies with a yearly phenoconversion rate of around 6% [22–24].

As a progressive disease, the symptoms of LBD continually worsen, and because LBD presents with great clinical and pathological heterogeneity, disease progression and presentation can vary significantly from person to person. This complex and variable clinical presentation along with the lack of a clinical diagnostic test make incidence and prevalence studies of LBD challenging and relatively rare. Yet, systematic reviews estimate the incidence rates of DLB to range from 0.5 to 1.6 per 1000-person-years, while the incidence rate of PDD is estimated to range from 3.91 to 4.99 per 100 person-years [25, 26]. Given these reported rates, highly penetrant variants would be expected to occur at very low frequencies within genetic databases. The typical age of disease onset is 70–85 years, making age the leading risk factor for LBD [4].

The older age at onset suggests that it is the interaction of genetic susceptibility and age-related changes that drives disease pathogenesis. Sex also seems to play a role in disease progression, as it is more common in men who tend to develop LBD earlier, have a longer disease duration, and present with key clinical diagnostic symptoms sooner than women, allowing for a quicker diagnosis after disease onset [6, 27–29]. Patients have been known to live with the disease for anywhere from two to twenty years, with the average disease duration lasting five to seven years [21]. As there are no specific treatments to slow or stop progression, it is currently an incurable disease.

Some therapies can be used to manage symptoms, but with the complexities of being a multi-system disease, each patient’s treatment plan will be different and often requires a comprehensive, multi-provider approach. Another factor adding complication to treatment planning is that some treatment options traditionally used to alleviate AD symptoms, such as anticholinergic, antidopaminergic, and traditional antipsychotic medications, can exacerbate LBD symptoms and speed the rate of mortality [30–33]. These adverse reactions demonstrate the dire need to determine the mechanisms driving LBD to help effectively design treatment and diagnostic methods. The current lack of available testing in the general population can often lead to a misdiagnosis, which can then significantly impact patient outcomes.

### α-synuclein pathology

Currently, the only way to definitively diagnose a patient who is presenting with symptoms of LBD is through an autopsy [4, 34]. Upon autopsy, Lewy body disease is characterized by the presence of Lewy bodies and Lewy neurites in the brain, with the clinical phenotype largely being determined by the regional distribution of this pathology [35]. In particular, the involvement of neocortical and limbic regions is strongly associated with cognitive impairment [35]. The propagation of α-synuclein pathology to these regions is what distinguishes DLB and PDD from PD, although concomitant pathologies can often blur this differentiation [36–38]. The end-stage pathology of DLB and PDD, however, does not differ, with the distinction between the two being a clinical classification. Both share defining features of diffuse Lewy body disease at autopsy and instead present as phenotypic variants along the Lewy body disease spectrum. The Lewy bodies and Lewy neurites that are seen at autopsy are formed by the aggregation of the protein α-synuclein. The protein exists normally in the brain of healthy individuals, particularly in presynaptic terminals, and while it is thought to play a role in synaptic function and neurotransmitter release, its exact purpose and function have yet to be determined [4, 21, 39, 40].

**Fig. 2:**
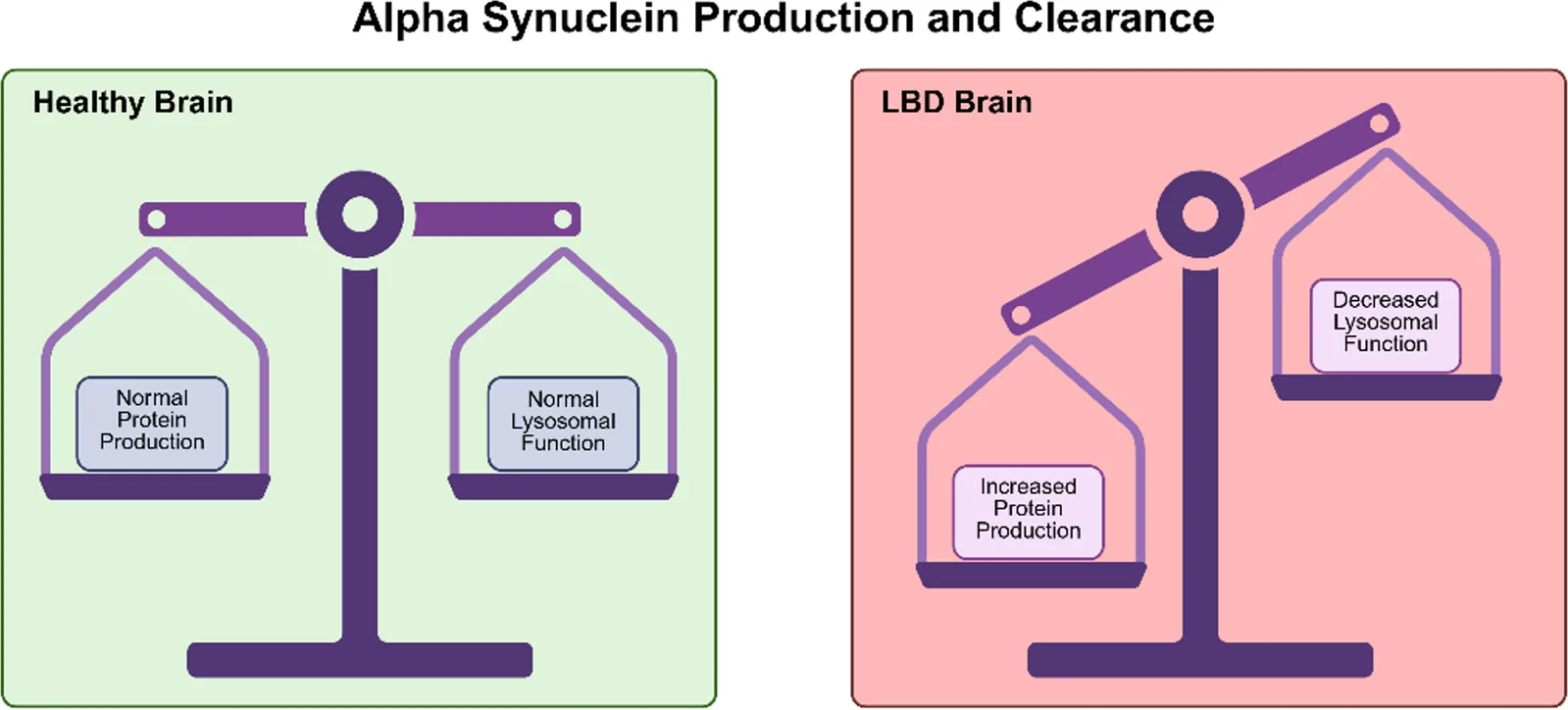
Imbalance between protein production and lysosomal function in LBD. In a healthy brain, protein production and lysosomal degradation are in balance, as shown by an evenly weighted scale. In contrast, in an LBD brain, an increase in protein production overpowers/outweighs the degradation ability of a dysfunctioning lysosome, which could be what is leading to Lewy body pathology. It is thought that the dual impact of α-synuclein overproduction and reduced degradation is what leads to the accumulation of the protein (Created in BioRender. Watkins, M. (2026) https://BioRender.com/zr09emm)

More specifically, LBD is also typically associated with significant degeneration of dopaminergic neurons in the substantia nigra, which leads to a reduction in dopamine levels and striatal communication, linked to both motor and cognitive dysfunction [41–44]. Additionally, cholinergic deficits, resulting in reduced levels of acetylcholine, have been found to contribute to cognitive dysfunction and neuropsychiatric symptoms like hallucinations and fluctuations in levels of cognition [44, 45]. It remains somewhat controversial whether the neurodegeneration associated with LBD is driven by the presence of Lewy body pathology, which leads to synaptic dysfunction and neuronal loss. However, the accumulation of the protein is believed to be driven by the overproduction of α-synuclein, improper clearance of the protein due to lysosomal dysfunction, or a combination of the two (Fig. 2).

### Co-pathologies

In addition to α-synuclein, LBD has significant pathological heterogeneity, with some studies indicating that between 70-80% of Lewy body disease cases have at least one co-pathology [46, 47]. With that, the majority of cases have AD co-pathology consisting of amyloid-β plaques and tau neurofibrillary tangles [8–12]. This co-pathology has been shown to have significant impacts on LBD, with many researchers demonstrating that it worsens cognitive decline and speeds the rate of mortality [10, 48, 49]. Interestingly, it is also much more common for patients with LBD to have a family history of other forms of neurodegeneration, such as AD, than a history of LBD. This was investigated in a 2020 study [50], comparing sporadic DLB patients to patients with a family member who was diagnosed with either PD or AD. It was found that the familial patients had significantly shorter survival than the sporadic patients. It was also noted that there were elevated cerebrospinal fluid (CSF) AD biomarkers in the familial DLB patients, which reiterates that AD co-pathology leads to a higher mortality rate. TAR DNA-binding protein 43 (TDP-43) pathology is also often prevalent in Lewy body disease cases and was recently found to play an important role in LBD misdiagnosis. In 2020, Buciuc et al. noted that patients with LBD who have hippocampal TDP-43 co-pathology are likely to receive a different clinical diagnosis, as it greatly alters disease presentation and prevents the typical LBD symptoms from showing [51]. Furthermore, TDP-43 presence has been linked to a more severe α-synuclein pathology, indicating a potential synergistic effect between the two proteins [52].

## Known genetic risk factors in LBD

**Fig. 3:**
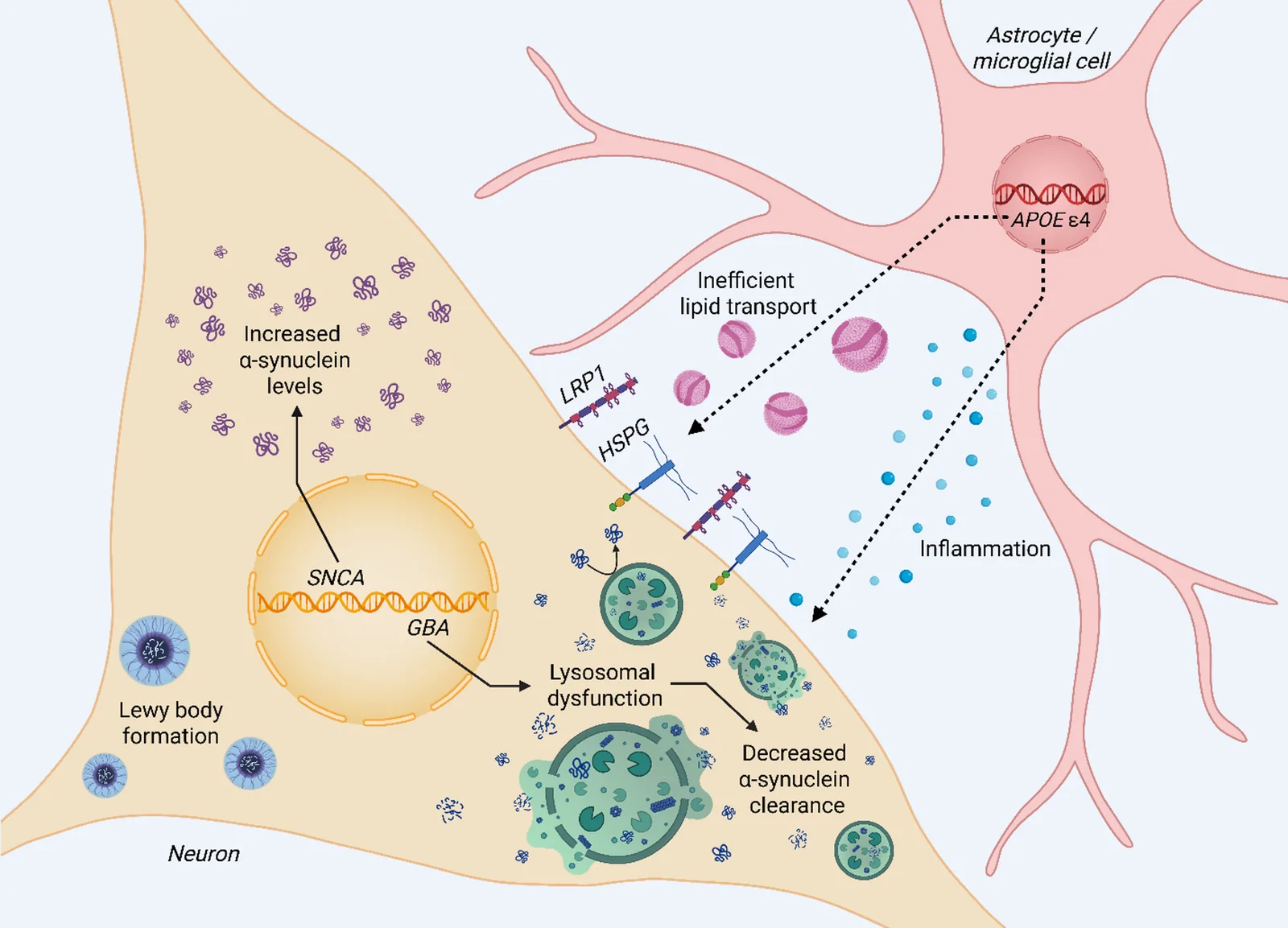
Genetic contributions to α-synuclein accumulation and LBD. Within the neuron, mutations or multiplications in *SNCA* lead to increased levels of α-synuclein, overwhelming the cell’s ability to process the protein. On the other hand, mutations in *GBA* impair lysosomal function, reducing the clearance of misfolded or excess α-synuclein. Meanwhile, *APOE ε4* disrupts cholesterol and lipid transport while promoting an inflammatory response, altering the clearance of α-synuclein and other proteins. Each of these genetic factors contributes to the formation of Lewy bodies through distinct pathways (Created in BioRender. Watkins, M. (2026) https://BioRender.com/ia6zdhl)

Research into LBD and related α-synucleinopathies, such as PD, has evolved considerably over the past few decades. Initially, LBD and PD were believed to be entirely sporadic disorders with no clear hereditary component. Early investigations therefore focused primarily on environmental triggers, such as toxins and viral infections, a view supported by low concordance rates in twin studies [53–55]. Although most cases remain sporadic, the identification of rare families with autosomal dominant inheritance led to a shift in focus toward familial forms. These discoveries, particularly the identification of highly penetrant mutations in genes such as *SNCA* (encoding the α-synuclein protein) [56–58], reshaped our understanding of LBD pathobiology and enabled the development of disease models and therapeutic strategies targeting its central pathological hallmark, α-synuclein. More recently, genome-wide association studies (GWAS) have expanded this understanding by identifying common genetic variants associated with LBD risk. A landmark GWAS published in 2021 confirmed three previously reported risk genes, *SNCA*, *APOE*, and *GBA*, and identified two novel loci, *BIN1* and *TMEM175* (Table 1) [17]. Notably, *APOE* and *BIN1* are well-established AD risk genes, while *SNCA*, *GBA*, and *TMEM175* are strongly associated with susceptibility to PD. This overlap underscores LBD’s unique position at the interface of AD and PD, raising important questions about whether LBD is a distinct neurodegenerative disorder or a hybrid that shares mechanistic pathways with both. At the same time, some major GWAS risk genes for AD and PD, such as *MAPT* and *TREM2*, were not associated with LBD in these studies, suggesting that while LBD shares genetic features with both diseases, it also has unique molecular drivers. Together, these discoveries highlight the complex and overlapping genetic architecture of LBD (Fig. 3) and reinforce the need for integrated approaches that combine common and rare variant analyses with mechanistic and functional studies. Such strategies will be essential for fully elucidating the etiology of LBD and identifying effective therapeutic targets.

**Table 1:** Key genes and variants that are associated with LBD

Gene	Discovery Method	Variant/Locus	Direction	Proposed Functional Consequence	Effect in LBD	Citation
*SNCA*	Linkage studies	p.A53T p.A30P p.E46K	Causal	Increased α-synuclein aggregation, toxic gain of function	Lewy body formation	Polymeropoulos et al. 1997 [59], Kruger et al. 2001 [57], Zarranz et al. 2004 [58]
	Gene mapping	Duplication Triplication	Causal	Gene dosage, increased α-synuclein expression	Increased Lewy body burden, triplication associated with severe phenotype and earlier onset	Singleton et al. 2003 [60], Chartier-Harlin et al. 2004 [61], Ibanez et al. 2004 [62]
	GWAS	rs7680557 rs7681440	Risk	Increased *SNCA* expression	Increased LBD risk	Guerreiro et al. 2018 [70], Chia et al. 2021 [17]
*APOE*	GWAS	ε4	Risk	Promotes α-synuclein aggregation and amyloid-β deposition	Increased LBD risk, more severe cognitive impairment	Sabir et al. 2019 [84], Chia et al. 2021 [17]
	GWAS	ε2	Protective	Reduced amyloid aggregation	Reduced risk of LBD	Sabir et al. 2019 [84]
	Sequencing	APOE3-Jac (p. V236E)	Protective	Reduced amyloid aggregation, enhances lipidation	Reduced risk of LBD	Liu et al. 2021 [78]
*GBA*	Candidate gene	p.L444P p.E326K p.N370S	Risk	Lysosomal dysfunction, impaired α-synuclein clearance	Increased LBD risk, increasedα-synuclein accumulation	Liu et al. 2022 [109]
	GWAS	rs2230288 rs35749011	Risk	Reduced GCase function	Increased LBD risk	Guerreiro et al. 2018 [70], Chia et al. 2021 [17]
*BIN1*	GWAS	rs6733839	Risk	Increased neurofibrillary tangle pathology	Increased LBD risk, possibly through AD related pathways	Chia et al. 2021 [17]
*TMEM175*	GWAS	rs6599388	Risk	Reduced lysosomal function	Increased LBD risk, impaired α-synuclein clearance	Chia et al. 2021 [17]

Through GWAS, *SNCA*,* APOE*,* GBA*,* BIN1 and TMEM175* have been identified as key modulators of LBD, with roles in disease risk, causation, and/or protection

### *SNCA* - α-synuclein at the heart of LBD

Up until 1996, researchers recognized that protein aggregation was a major pathology of Lewy body disorders, but had no clear understanding of the specific drivers of disease. At that time in Contursi, Italy, neurologists were following a large pedigree with multiple affected members with a severe form of PD, with dementia as an early feature [56]. Through classical gene linkage (mapping) approaches, they identified a shared chromosomal region among affected members and subsequently a novel DNA mutation in the *SNCA* gene (encoding α-synuclein) [59]. The mutation resulted in a substitution, p.A53T, and is proposed to increase the aggregation propensity of the protein. Over the next few years, large-scale screening of the gene in Lewy body patient series demonstrated that *SNCA* mutations were relatively rare, with additional substitutions, p.A30P, found in a German family with PD, and p.Ε46K, found in a Spanish family with DLB, identified throughout the next decade [57, 58].

A paradigm shift in our understanding of disease mechanisms occurred in 2003 with a discovery in a large multi-incident US pedigree. Gene mapping in the family, known as the Iowa kindred/Spellman–Muenter kindred, with autosomal dominant inheritance of early-onset PD and rapid dementia onset, indicated the *SNCA* locus [60]. Autopsy of affected family members confirmed the presence of Lewy bodies, but their initial mutation screening of the *SNCA* gene was negative. Upon further examination, they discovered that the region surrounding the *SNCA* gene was within a large genomic triplication event resulting in 4 gene copies [60]. Subsequent screening further identified patients with familial PDD and *SNCA* triplication; crucially, patients were also identified who harbored duplication of the *SNCA* gene (3 gene copies), with a less severe clinical phenotype and later age-at-onset [61, 62]. This led to the hypothesis that over-expression of the gene is quantitatively correlated with a worsened disease phenotype, suggesting that an abundance of wild-type α-synuclein has a significant effect on disease progression and presentation. Over time, groups continued to screen families for *SNCA* multiplications, and it was noted quickly that they were a rare cause of disease [61–64]. These discoveries helped to elucidate that there was a dose-response between the number of *SNCA* copies and the disease severity and onset [65]. From these studies, it became evident that *SNCA* plays a central role in both the development of familial LBD and also the progression of disease.

Not only did this information provide a better understanding of potential disease mechanisms at play, but it has also offered a potential target for disease treatment. It is hoped that *SNCA* knockdown will reduce the amount of misfolded α-synuclein protein accumulation. Some studies have already begun in this field, and while they have shown promising results in the early pre-clinical stage development, there is still much to be done before in-human clinical trials can be considered [66]. Other studies have leveraged the identification of the *SNCA* triplication to develop models that can be used to further test therapies and gain a better understanding of disease development, such as the recent generation of iPSC-derived LBD organoids [67]. At first, it was thought that these types of studies and therapies would not be beneficial to the general population of patients with LBD, as although it is the second most common form of dementia, it is very rare to see Mendelian inheritance. However, as researchers began transitioning to population studies, the broader value of targeted α-synuclein therapies in non-familial patients was brought to light.

In recent years, common genetic variation studies have become a key component in our understanding of LBD and other complex neurodegenerative diseases. We have already seen great success in their implementation in AD and PD, which has set a precedent for similar research in LBD [68, 69]. GWAS, one of the most comprehensive population-based genetic studies, examines the relationship between common genetic variation and specific disease phenotypes in an unbiased manner. With *SNCA* being identified as a risk factor for LBD through familial studies, its importance not only in familial but also sporadic LBD was confirmed when it was identified as one of the top hits in an LBD GWAS. The study included 1,743 patients diagnosed with LBD, 1,324 of whom also had a neuropathological diagnosis, and 4,454 controls. The GWAS highlighted the association of *SNCA*,* APOE*, and *GBA* variants with disease risk, all confirmed in the study’s replication series [70]. A subsequent GWAS was performed two years later with 828 clinically diagnosed DLB patients and 82,035 controls confirming the results, identifying the same three genes as risk loci [71]. From both of these population studies as well as the familial studies, it is known that *SNCA* plays a significant role in both LBD and PD development, with it being one of the top GWAS hits for both diseases. However, it is noteworthy that the top *SNCA* GWAS variants for each condition were switched [70]. This is especially important as in 2005, it was found that 24% of newly diagnosed PD patients showed signs of dementia at diagnosis, and upwards of 80% of PD patients will develop dementia, demonstrating the fine line between PD and LBD [16, 72]. Notably, studies in PD have highlighted a role for *GBA* in cognitive progression, thus, like *SNCA*, it is both a disease risk and phenotype modifying locus [73]. However, studies have also associated the *APOE* locus, not a recognized PD susceptibility factor, with composite and cognitive progression in PD [74, 75]. With this, although α-synuclein is central to LBD, both at the genetic and protein level, the top variant hit in the population-based GWAS is actually at the *APOE* locus.

### *APOE* – a link between LBD and AD

The apolipoprotein E (*APOE*) gene, located on chromosome 19, encodes a crucial lipid transport protein involved in cholesterol metabolism as well as essential functions such as neuronal repair and inflammation in the central nervous system [76]. There are three common *APOE* alleles, ε2, ε3, and ε4. Among these, the ε4 allele is the strongest known genetic risk factor for late-onset AD, while the ε2 allele is generally considered protective, compared to the neutral ε3 allele [76]. Indeed, the *APOE* protective variant (APOE p. V236E; *APOE3*-Jac) that was identified to protect against AD, was also shown to be protective against LBD [77–79]. The role of *APOE* in LBD was long debated; however, recent GWAS have firmly established *APOE* ε4 as the major common genetic risk factor for LBD, with an odds ratio of 2.46 (95% CI: 2.22–2.74) and an allele frequency of 10% in controls versus 21.3% in LBD cases [17]. A critical question remains: does the *APOE* ε4 allele (and related AD loci, e.g. *BIN1*) influence LBD risk primarily through coexisting Alzheimer’s pathology, or does it independently drive α-synuclein pathology?

Several postmortem studies have sought to disentangle this relationship. An early study reported that *APOE* ε4 was associated with dementia across multiple groups, including “pure” DLB, PDD, and AD cases, suggesting an ε4-driven risk even in the absence of significant amyloid pathology [80]. A larger autopsy-based study led by our group later found that *APOE* ε4 was linked to greater Lewy body burden, independent of AD co-pathology, across five brain regions [81]. Similarly, a recent GWAS of neuropathological traits reported that ε4 carriers had increased levels of plaques, tangles, and Lewy bodies [82]. At the molecular level, *APOE* ε4 exacerbates dysfunction in LBD brains compared to *APOE* ε3, with increased *SNCA* expression and pronounced layer-specific transcriptional alterations affecting synaptic and bioenergetic pathways, as shown by recent spatial transcriptomic analyses of postmortem LBD brains [83]. In contrast, *APOE* ε2 may be protective against LBD [84]. However, not all findings are consistent. For example, Kaivola et al. reported that ε4 was associated only with LBD cases that also had intermediate or high levels of AD pathology, but not with pure LBD cases [85], a result validated by another recent study [86]. These conflicting findings raise the possibility that *APO*E ε4’s effects on α-synuclein pathology may be mediated, at least in part, through its influence on amyloid deposition.

Given the high prevalence of coexisting AD and Lewy body pathologies in human brains, it remains challenging to tease apart the effects of *APOE* on α-synuclein pathology from its well-established role in amyloid deposition. However, animal and cellular models provide valuable systems to study α-synuclein-related mechanisms in isolation. To explore this, our group and others have used mouse models expressing human *APOE* isoforms. These studies found that *APOE* ε4 exacerbated α-synuclein pathology even in the absence of amyloid, and was associated with worsened motor performance, increased synaptic loss, and heightened neuroinflammation [87, 88]. Transcriptomic analyses revealed that *APOE* ε4 disrupted lipid metabolism, protein homeostasis, and neuronal signaling pathways [87], supporting a direct role in α-synuclein aggregation and toxicity. Moreover, *APOE* ε4 mice showed increased propagation of α-synuclein following intrastriatal injection of preformed fibrils (PFFs) [88], pointing to enhanced spread of pathology in vivo. Complementary findings have emerged from human iPSC-derived cortical organoids. Organoids lacking *APOE* or expressing the ε4 isoform showed increased accumulation of insoluble α-synuclein, reduced expression of synaptic markers, and abnormal lipid droplet accumulation. Strikingly, these pathological features were rescued by treatment with astrocyte-derived *APOE* ε2 or ε3, but not ε4, highlighting isoform-specific differences in lipid regulation and α-synuclein clearance [89].

It is well established that α-synuclein pathogenesis involves two key processes: seeding and propagation. Jin et al. employed real-time quaking-induced conversion (RT-QuIC) assays and found significantly greater α-synuclein seeding activity in brain samples from *APOE* ε4 carriers with both AD and LB pathology, and importantly, also in “pure” LBD cases with minimal amyloid burden [90]. When these amplified a-synuclein seeds were applied to iPSC-derived neurons, seeds from *APOE* ε4 induced greater toxicity and reduced neurite outgrowth, suggesting that aggregates from *APOE* ε4 carriers are more neurotoxic, even without AD pathology. Although it has not yet been directly tested whether *APOE* ε4 influences α-synuclein propagation per se, emerging evidence points to a potential mechanism. The LDL receptor-related protein 1 (LRP1), a major receptor for the APOE protein, was recently found to regulate α-synuclein uptake in neurons [91], suggesting that APOE could also modulate intercellular propagation of pathology, an area warranting further study.

Interestingly, despite its strong involvement in LBD and AD, *APOE* has not been identified as a risk factor for PD in GWAS [92]. However, *APOE* ε4 does appear to influence the progression from PD to PDD [93, 94]. *APOE* ε4 carriers experience more rapid cognitive decline [88, 95], earlier onset of dementia symptoms [96], and an age-dependent increase in risk for cognitive deterioration [97]. Notably, these effects seem to be limited to cognitive domains and do not appear to impact motor progression, suggesting that *APOE* ε4 contributes specifically to cognitive vulnerability in α-synucleinopathies.

**Fig. 4:**
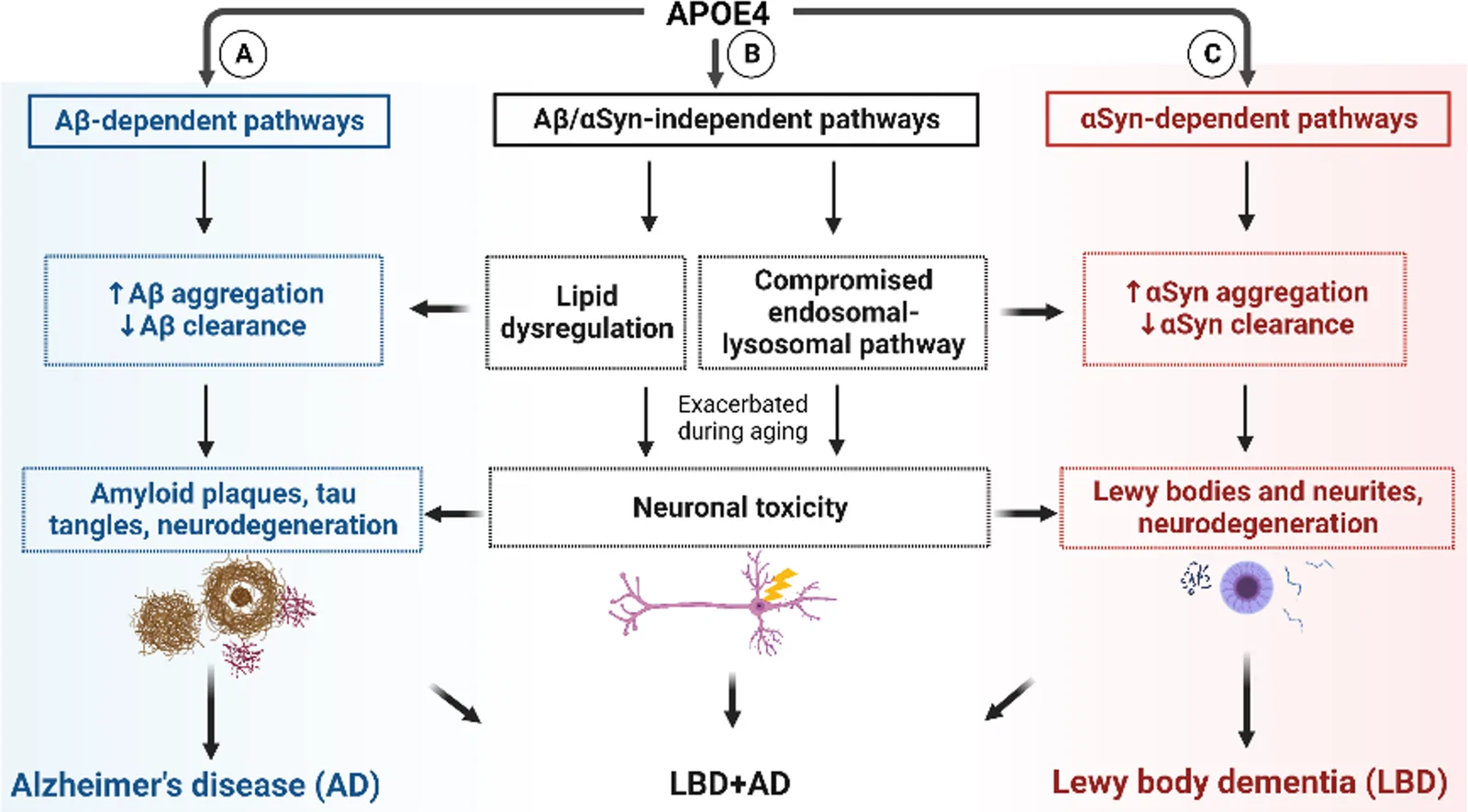
Graphic summary of how *APOE* ε4 may influence multiple pathways in the pathogenesis of AD and LBD. *APOE* ε4 is proposed to modulate AD and LBD pathogenesis through amyloid-β (Aβ)-dependent pathways (**A**), α-synuclein (αSyn)-dependent pathways (**C**), and Aβ/αSyn-independent pathways (**B**). In Aβ-dependent pathways (**A**), *APOE* ε4 strongly promotes Aβ seeding and aggregation while impairing Aβ clearance, leading to amyloid plaque accumulation, tau tangle formation, and neurodegeneration. In αSyn-dependent pathways (**C**), *APOE* ε4 may enhance αSyn seeding and aggregation and reduce αSyn clearance, contributing to Lewy body and Lewy neurite formation and subsequent neurodegeneration. In Aβ/αSyn-independent pathways (**B**), *APOE* ε4 may disrupt lipid metabolism and impair endosomal-lysosomal function, processes that can be exacerbated by aging, thereby increasing neuronal toxicity and contributing to disease progression independently of Aβ or αSyn pathology (Created in BioRender. Xhafkollari, G. (2026) https://BioRender.com/uw2p5a3)

Together, these findings illustrate the multifaceted role of *APOE* in LBD. While some effects may be mediated indirectly through AD co-pathology, converging evidence from animal models, human postmortem tissue, and stem cell systems support a direct role for *APOE* ε4 in promoting α-synuclein aggregation, spread, and neurotoxicity (Fig. 4). These data underscore the importance of lipid biology in LBD and point to *APOE*-related pathways as promising targets for therapeutic development.

### *GBA* – glucocerebrosidase and the lysosomal link

*GBA* encodes the lysosomal enzyme beta-glucocerebrosidase (GCase), which has a primary role of breaking down glucosylceramide (GlcCer), a glycosphingolipid, into glucose and ceramide [98]. Loss-of-function biallelic *GBA* mutations cause Gaucher disease, a recessive lysosomal storage disorder [99]. While studying Gaucher disease, astute researchers observed that many of the patients had a family history of parkinsonism [100–103]. These affected relatives were observed to carry heterozygous *GBA* mutations, which aided in the discovery that monoallelic mutations, likely resulting in decreased enzyme activity, are linked to an increased risk of PD and LBD [103–107]. Notably, mutations that are causative in Gaucher disease are also associated with risk of LBD, however, there are also other frequent *GBA* variants that increase risk. Although the frequency of *GBA* mutations in LBD can be relatively high, with estimates up to 15–20% in populations of Ashkenazi Jewish descent [108], multiple different mutations account for the population risk. Indeed, given the rare nature of *GBA* mutations, gene burden approaches are necessary to combine mutations across patient-control series for association studies. In the recent genome sequencing study, Chia et al. performed global gene burden analysis across genes, and *GBA* was the only gene locus to associate with LBD risk [17]. Moreover, the GWAS analysis also highlighted an association with common variation and the frequent coding variant that results in a p.E326K substitution.

LBD *GBA* mutation carriers tend to have an intensified disease presentation with more severe cognitive and motor symptoms and an earlier age of onset compared to non-carriers [106, 108–113]. Additionally, they exhibit more frequent visual hallucinations as well as RBD and were noted to have less severe AD-related pathology [108, 114]. The combined frequency of rare *GBA* mutations and common disease risk factors, together with its relevance as a rational therapeutic target, results in *GBA* being recognized as one of the most clinically relevant genetic loci in LBD. Thus, the association between *GBA* and LBD is likely due to a breakdown of a very specific lysosomal function. Lysosomes play a key role in α-synuclein degradation, and mutations within *GBA* likely lead to impaired degradation of the protein. This promotes accumulation and aggregation of α-synuclein, which in turn increases risk of developing LBD or PD. Thus, similar to APOEε4, *GBA* does not specifically segregate with LBD phenotypes in families, but in general, acts as a risk factor that increase the likelihood of developing LBD. However, it is important to acknowledge that the identification of genes that influence risk, irrespective of effect size, can provide a window into the underlying mechanism of disease.

A clearer understanding of GCase function in LBD came through early work completed in primary culture neurons expressing human α-synuclein, outlining a feedforward loop as the potential mechanism through which α-synuclein and GCase drive each other’s pathogenicity [115]. Knocking down GCase in neurons resulted in decreased α-synuclein clearance and an accumulation of α-synuclein in lysosomes with no overall effect on total α-synuclein levels in the cell. The study also found that the addition of GlcCer to recombinant α-synuclein in acidic conditions stabilized the formation of higher molecular weight α-synuclein, which in turn caused an accumulation of GCase in the endoplasmic reticulum (ER) and lower levels of glycosylated post-ER GCase. This subsequently caused lower levels of lysosomal GCase activity, resulting in the accumulation of GlcCer in lysosomes, which cannot be hydrolyzed. This accumulated GlcCer then stabilizes the formation of α-synuclein oligomers, promoting their conversion to fibrils. The α-synuclein aggregation also blocks GCase from being shuttled into the lysosomes, which elevates GCase’s accumulation in the ER, further promoting low GCase activity, accumulation of GlcCer, and the stabilization of soluble α-synuclein oligomers, reinforcing the pathogenic cycle [115].

The work that followed focused on evaluating the effect of GCase and specific *GBA* mutations in specific cell populations. Some groups utilized an in vivo approach to knock out *GBA* primarily in neurons. Deleting *GBA* in cortical neurons and glial precursor cells resulted in rapid motor dysfunction and ultimately paralysis at postnatal day 21 with microglial activation, increased cytokine and chemokine levels, and blood-brain barrier integrity impairment at this time point [116]. Focusing more specifically on myelinating glia, deletion of *GBA* in oligodendrocytes showed reduced protein levels of myelin-related proteins and consequently impaired myelination, as observed by electron microscopy [117]. *Gba* p.E326K mice at 24 months old show motor impairment paired with loss of TH+ neurons in the substantia nigra and an overall reduction in neurons and gliosis in the hippocampus. However, the effects on α-synuclein pathology remain limited in these models without the addition of an α-synucleinopathy model. For example, in the *Gba* p.E326K mice, it is only when α-synuclein pre-formed fibrils are introduced in the gut of these mice that α-synuclein pathology is seen in the hippocampus; others have observed similar results [118, 119]. A study utilizing GBA p.D409H mice crossed with human p.A53T α-synuclein transgenic mice saw earlier and increased α-synuclein pathology, paired with a loss of dopaminergic neurons and heightened gliosis in the midbrain in comparison to control human p.A53T α-synuclein transgenic mice [120]. A similar effect was noted in *Gba* p.L444P mice receiving human α-synuclein via AAV injections in the midbrain [121]. Overall, studies done in vivo have shown modest effects on α-synuclein pathology in mice solely carrying *Gba* mutations or knockout. However, it is important to note that the addition of these mutations or *GBA* knockout to synuclein models further impairs α-synucleinopathy and promotes loss of dopaminergic neurons in the midbrain, pointing towards an important contribution of GCase to pathology. These studies pave the way for a broader understanding of the α-synuclein-GCase axis in LBD and highlight the importance of *GBA* variation for diagnosis, treatment, and future research.

In summary, *SNCA*, APOE, and *GBA* have been demonstrated to be key genetic determinants of LBD, highlighting how disruption of α-synuclein homeostasis, altered lipid metabolism, and impaired lysosomal clearance drive disease pathogenesis (Fig. 3).

## The “imperfections” in LBD genetics

**Fig. 5:**
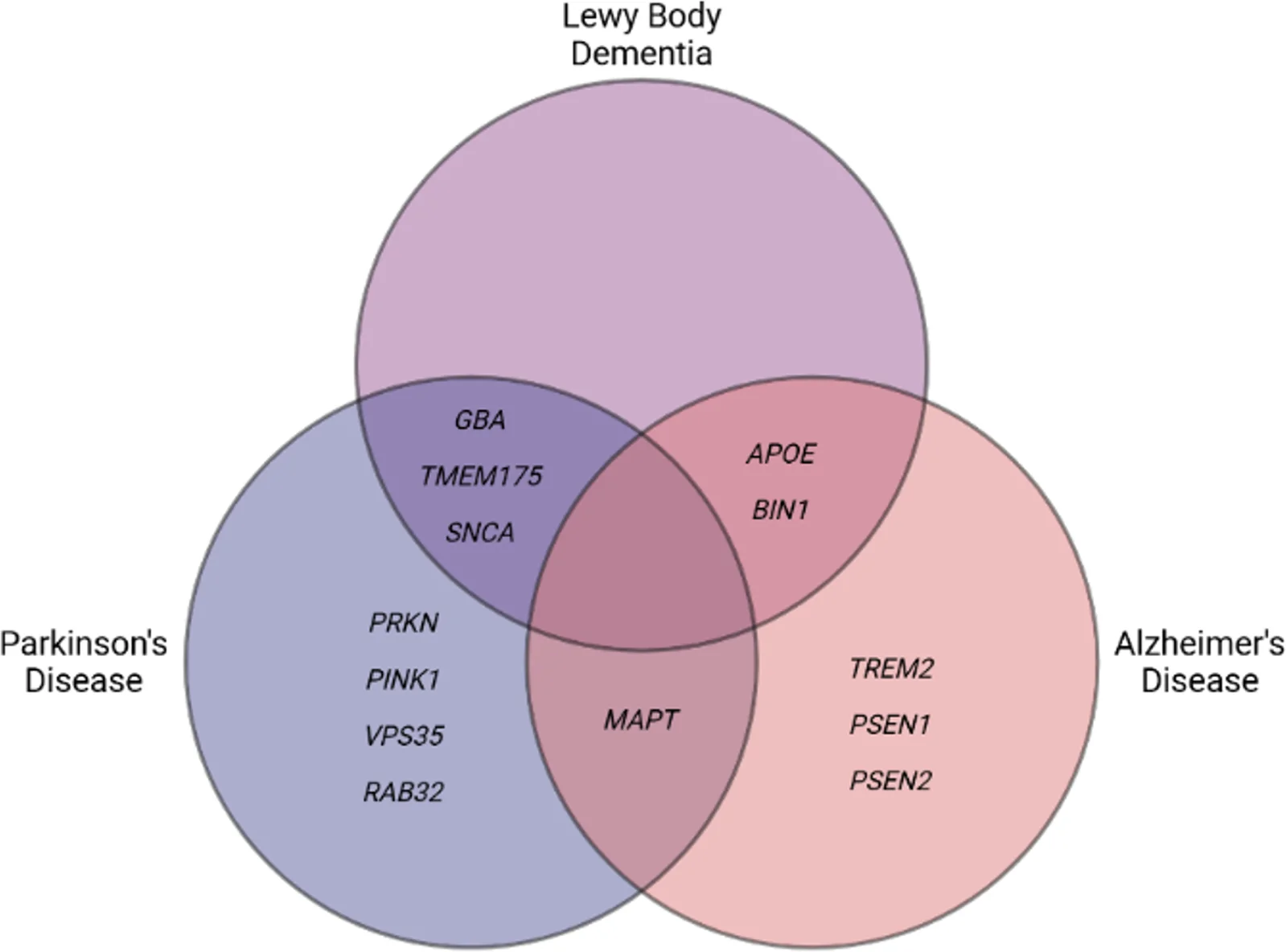
Genetic overlap and distinction between LBD, PD, and AD. Although LBD shares clinical and pathological features with PD and AD, its genetic profile reveals a more complex relationship. Genes associated with LBD are found to also be risk factors for either PD or AD, but notably, no genes have been identified to be associated only with LBD. This missingness may support the idea that LBD lies at the intersection of PD and AD rather than presenting as its own unique disease. However, PD and AD share an associated risk not seen in LBD, contradicting LBD being a genetic blend of PD and AD and suggesting there is a distinct pathogenic process at play *(*Created in BioRender. Watkins, M. (2026) https://BioRender.com/qpx2j4o)

LBD represents a complex, heterogeneous cognitive profile that appears to encompass features of more common motor (PD) and cognitive (AD) disorders but also displays distinct characteristics. The genetic understanding of LBD has also demonstrated a clear overlap with the established genetics of PD and AD, however not all well-established genetic risk is apparent in LBD (Fig. 5). For example, studies to date have not observed a disease association with *TREM2* variants, a strong AD risk factor, or at the *MAPT* locus, a strong genetic risk for both AD and PD [122, 123]. This may be evidence for the hypothesis that LBD is not just simply sitting along a spectrum of neurodegeneration between PD and AD, or that it simply presents when a patient has a combination of the two diseases. Other genetic factors have been implicated in LBD that may reflect shared biology or mixed pathologies across neurodegenerative dementias, such as *GRN* mutations, a relatively common cause of frontotemporal dementia (with TDP-43 pathology), and a potential rare cause of LBD [124]. These findings have implications for disease intervention trials with combination therapies, each targeted at specific protein pathologies, required to address the full gamut of LBD.

To date, no single gene has been identified as accounting for the majority of LBD cases, highlighting that even with current technologies, there is still something important we are missing about the underlying genetics driving the manifestation of LBD. Our genetic understanding of LBD is still in its infancy, and the need for greater clinical recognition of the disorder and focused sample collection will be imperative to gain further insights. It is notable that the most recent GWAS efforts in LBD included 2,591 patients with LBD, whereas the latest AD and PD efforts both included over 60,000 patients each [68, 69]. Thus, we need to start now with standardized clinical characterization and biospecimen collection protocols to reach the necessary sample numbers to truly resolve the underlying genetic etiology of LBD. However, building accurate and well-defined LBD research cohorts remains challenging. Clinical heterogeneity and overlapping symptoms make it difficult to assemble consistent clinical cohorts, while even carefully defined pathological cohorts are often complicated by the frequent presence of co-pathologies which can often blur distinctions between diseases.

While it is well known that α-synuclein is central to LBD pathology, reliable in vivo biomarkers are still under development. In clinical practice, diagnosis remains heavily reliant on clinical criteria and symptomology, which are often variable and subjective due to the disease’s heterogeneity. LBD presents as a broad spectrum of phenotypes, with cognitive, motor, and neuropsychiatric features often resembling those of other neurodegenerative diseases [6]. Additionally, the order and prominence in which these symptoms appear varies greatly. This non-linear and non-specific progression complicates diagnosis and classification, leading to high levels of misdiagnosis, affecting everything from clinical care to drug development. These diverse disease features complicate clinical trial design and analysis, undermine prognostication, hinder accurate and early delivery of treatments and therapies, and lead to an overall higher caregiver and patient burden surrounding the uncertainty of a frequently shifting diagnosis [125]. Improving the phenotyping of LBD is a critical step towards unraveling the complexities of its diagnosis. Enhanced technologies (e.g. sleep/actimetry sensors, heartrate monitors, accelerometers, blood pressure monitors, glucose monitors, etc.) that are able to help identify the symptoms patients and their families don’t often observe and report on their own, such as heartrate, activity levels, and sleep patterns, could help to provide a more real-time characterization of the disease features caused by genetic mutation. At the same time, biomarker development is rapidly advancing. Seed amplification assays (SAA), which detect misfolded α-synuclein, are showing potential to be a promising diagnostic tool capable of detecting Lewy body pathology in early stages of disease, with the hope of being able to detect pathology even before symptoms begin [126–128]. Given these diagnostic difficulties, making progress in our ability to diagnose and treat LBD will require the use of emerging biologically grounded technologies.

## Emerging insights and technologies

Genetic sequencing technologies have greatly advanced over the last decade. With the advent of next-generation methodologies, whole-genome sequencing has become a standard approach to identifying genetic variation. A GWAS on 5,213 samples with non-Alzheimer’s dementias (LBD and FTD/ALS) and 4,132 controls was able to use short-read whole genome sequencing data to identify a 309-base pair (bp) deletion within *TPCN1* that was significantly associated with the LBD cases in their cohort [129]. This discovery was made using a multi-algorithm pipeline specifically designed to detect structural variation (SV), a type of variation often difficult to resolve. However, despite recent advances, traditional short-read approaches (100–300 bp long fragments) and the inherent difficulties that come with mapping them back to the genome have resulted in large regions of the genome unresolved; this is particularly evident in repetitive regions as well as areas containing other complexities [130]. To improve our understanding of LBD genetics, the use of long-read sequencing technologies will allow researchers to achieve much longer sequencing reads that are between 10 and 20 kbp [131]. This innovative approach has great potential in detecting variation not identified by conventional short-read sequencing, including large complex SVs (e.g. copy number variations (CNVs), deletions, inversions, repeats, etc.). Using long-read sequencing makes it much easier to sequence areas of the genome that are considered too complex/difficult for short-read sequencing to accurately capture, like GC-rich areas and highly repetitive regions [132]. The application of long-read sequencing approaches will allow for further discrimination of the variants that may be driving this disease. Through a recent GWAS of atypical frontotemporal dementia with ubiquitinated inclusions (aFTLD-U), a major risk locus was identified on chromosome 15q14 [133]. To further explore this finding, the study employed the use of long-read sequencing and identified a tandem repeat expansion within *GOLGA8A*, present in almost 60% of the cases tested, demonstrating the unique capabilities of this powerful sequencing method [133]. Indeed, an unresolved genome-wide linkage analysis in LBD identified a novel disease-linked region on chromosome 2q35-q36 in a Belgian family (DR246) with clinical and pathological DLB. Sequence analysis of the exons within a 9.2 Mb region, however, did not identify any pathogenic mutations [134]. Subsequently, more members from the family began to present with symptoms, and a wider analysis was performed, shrinking the locus size from 9.2 Mb to 3.3 Mb; still, no disease-causing mutations were found [135]. These efforts may benefit from long-read sequencing within the targeted region to help resolve the pathogenic mutation if present.

At the population level, increased sample sizes will drive up the number of GWAS significant loci, as we have witnessed for PD and AD; however, it will be important to consider how these genetic determinants will fundamentally shape our understanding of LBD etiology and help aid in the development of both diagnostic and therapeutic tools. Given the broad nature of GWAS approaches it will be critical to determine the disease-related genes in nominated loci, genomic fine-mapping approaches (e.g., colocalization/eQTL integration, transcriptome (TWAS), functional annotation) can not only strengthen the evidence of association but also help the interpretation of association signals and define disease genes/variants. The incorporation of such genetic factors into the diagnostic arsenal of the neurologist remains to be truly realized, as each individual locus of relatively ‘weak’ effect size does little to inform into either an accurate diagnosis or prognostic outlook. Current genetic approaches to generate polygenic risk scores (PRS) provide a measure of overall genetic susceptibility, but their utilization is limited. The incorporation of disease relevant PRS relies upon the identification of individually associated-loci, and for LBD, those need to be identified. However, the application of AD and PD PRS may help discriminate between LBD subtypes and guide physicians on who may benefit most from therapies targeted at these related disorders. Finally, while genetic studies have allowed us to identify numerous risk loci, their functional consequences remain unclear. Transcriptomic approaches, such as single-nucleus RNA-seq (snRNA-seq), would provide cell type specific expression of these risk factors, while the incorporation of other multi-omic datasets (epigenomics, proteomics, etc.), along with the development of artificial intelligence and machine learning methods, will provide a more nuanced way to view ‘LBD’ as we move into the future.

In the meantime, perhaps we can glean a clearer pathomechanistic picture through the functional studies targeted at the genetic factors highlighted in this review. α-synuclein remains the central focus of LBD, and incorporating the other major genetic risk factors like APOE and *GBA* is critical, as they may further exacerbate α-synuclein levels/pathology and the phenotypes associated with it, including neuroinflammation, neurodegeneration, gliosis, and subsequent impacts. These findings remain at the phenotypic level of pathology, as it is still unclear how these proteins interact and influence each other at a cellular level. While the potential mechanism for how α-synuclein and GCase can interact and drive pathology has been outlined in neurons, it is unclear whether the same interaction also occurs in other cell types. Importantly, what remains to be elucidated is the mechanisms through which APOE ε4, a lipoprotein that is synthesized and released primarily by glia, can affect α-synuclein pathology seen in neurons. As such, future work should focus on how these interactors come together to promote pathology while considering cell-to-cell interactions. Additionally, α-synuclein function, aggregation, and clearance should be explored in other non-neuronal cell types to gain insight into disease mechanisms acting independently of neuronal dysfunction. As such, studies focusing on pathway and network analyses are needed to identify additional contributors that can then be explored in future studies. Such work can also lead to the identification of novel therapeutic targets, ultimately focusing on slowing down the exacerbation of pathology.

Even with the gaps in our knowledge of LBD etiology, there are potential therapeutic strategies that can be followed. Given the well-established genetic links with α-synuclein overexpression as a driver of LBD susceptibility and phenotypic modification, approaches to reduce levels are significant avenues of intervention. Whether it be at the ‘production’ levels using antisense oligonucleotides, silencing RNAs, etc., or at the clearance level, with potential immunodepleting therapies, there is great promise across the spectrum of Lewy body disorders. Genetics of LBD and lessons from the Gaucher disease field suggest that a brain-penetrant enzyme replacement therapy may benefit those with genetic mutations, but upregulation may help lower α-synuclein generally and provide a broader treatment approach across patients with LBD regardless of *GBA* mutation status. Finally, APOE-targeted therapies may offer dual benefits for both LBD and AD. Several strategies are currently being explored in the AD field, including reducing APOE expression, particularly in ε4 carriers, to mitigate its toxic gain-of-function effects in neurodegeneration. Enhancing *APOE* lipidation, for example through activation of lipid transporters such as ABCA1, may improve its neuroprotective functions and facilitate the clearance of toxic proteins. Gene replacement or editing approaches, such as converting the ε4 allele to the more protective ε2 form, are also under investigation using CRISPR and related technologies. Although these strategies have shown promise in AD, they remain largely unexplored in the context of LBD and warrant further investigation. Additionally, targeting APOE receptors such as LRP1 may help regulate the propagation of not only α-synuclein, but also amyloid and tau pathology. These approaches, while still in preclinical or early clinical stages, reflect the growing recognition that APOE is not just a genetic risk factor but a modifiable driver of disease, with therapeutic relevance across both AD and LBD.

In addition to disease risk, identifying phenotypic modifiers will be crucial in improving our understanding of potential therapeutic intervention strategies in LBD. Many recent research efforts have focused solely on identifying risk factors for LBD to better understand its etiology. However, little has been done to identify phenotypic modifiers of this disease. These variables influence how LBD manifests in individuals who are at risk and alter disease presentation and course. Phenotypic modifiers could include genetic, environmental, or lifestyle factors that influence not only whether a person develops LBD but also variables like their age of onset, disease progression, the severity of their symptoms, and the level of degeneration in the cells in their brain. Given the evidence from genetic studies, factors such as coffee drinking, considered protective in PD, and exercise, protective across neurodegenerative disorders, are likely to impact LBD risk. At present, however, we lack a complete understanding of the biological and environmental factors that interact with genetic risk to shape the clinical outcomes of LBD. It is also critical across genetic, environmental, or lifestyle studies that we include diverse populations, as not only will factors be different across populations, but multi-ancestry genetic approaches will help guide gene and variant discovery in LBD, as was recently demonstrated in PD [92]. Patients of European, East Asian, Latin American and African ancestry not only identify novel loci but also helped refine/fine-map the potentially causal variant at six specific loci. Thus, the importance of identifying and understanding the heterogeneity of disease-related genetic determinants across populations will be critical in assessing environmental or lifestyle modifiers. Unfortunately, these studies lag far behind in LBD, but perhaps the lessons that have been learnt in parallel efforts in PD and AD will accelerate progress in LBD, and we can benefit from large-scale global collaborative initiatives such as the Global Parkinson’s Genetics Program (GP2) and the International LBD Genomics Consortium. These large consortia will be needed to uncover genetic, environmental, or lifestyle interactions and to better understand why some individuals with similar genetic predispositions experience rapid, severe symptoms while others have slower progression of disease. Research into phenotypic modifiers is essential for improving our ability to predict disease course, personalize treatment strategies, and ultimately develop effective treatments and therapies designed to suit each patient.

## Conclusion

Genetic studies of LBD have already provided a basis of knowledge on the underlying pathways driving disease susceptibility and progression. It remains to be resolved if the shared genetic profile of LBD with more frequent disorders PD and AD point towards a common underlying pathomechanism with core clinical and pathologic features, or a standalone genetic entity with symptomatic overlap. The combination of phenotypic and biomarker data with genetic information will help us to stratify patients for future studies and clinical trials, improve individualized treatment strategies, and pave the way for disease-modifying interventions. Ultimately, it is likely that it is the genetic ‘imperfections’ that will guide disease understanding and help end the battle against LBD.

## Data Availability

Not applicable.

## Abbreviations

LBD: Lewy body dementia
DLB: Dementia with Lewy bodies
PDD: Parkinson’s disease dementia
AD: Alzheimer’s disease
PD: Parkinson’s disease
REM: Rapid eye movement
RBD: REM sleep behavior disorder
CSF: Cerebrospinal fluid
TDP-43: TAR DNA-binding protein 43
GWAS: Genome wide association study
SNP: Single nucleotide polymorphism
PFF: Preformed fibrils
RT-QuIC: Real-time quaking-induced conversion
LRP1: LDL receptor-related protein 1
GCase: Beta-glucocerebrosidase
GlcCer: Glucosylceramide
ER: Endoplasmic reticulum
bp: Base pair
SV: Structural variation
CNV: Copy number variation
aFTLD-U: Atypical frontotemporal dementia with ubiquitinated inclusions
PRS: Polygenic risk score
SAA: Seed amplification assay
GP2: Global Parkinson’s Genetics Program
